# Resuscitation of a case of cardiac arrest complicated by resection of a giant mediastinal tumor: a case report and literature review

**DOI:** 10.3389/fonc.2025.1545496

**Published:** 2025-04-10

**Authors:** Mu Jie Ka Sha, Ling Huang, Chen yi Xiong, Chun yuan Zhao

**Affiliations:** Guangxi Medical University Cancer Hospital, Nanning, China

**Keywords:** giant mediastinal tumor, cardiac arrest, resuscitation, surgical removal, case report

## Abstract

This case report describes the successful resuscitation of a 54-year-old female who experienced cardiac arrest following resection of a giant mediastinal tumor (GMT). The patient’s hemodynamic collapse was attributed to mediastinal mass syndrome, mediastinal swing, and acute re-expansion pulmonary edema. This report highlights the challenges of perioperative management in GMTs, emphasizing the importance of multidisciplinary preoperative planning, continuous hemodynamic monitoring, and prompt intervention during complications. Early recognition of hemodynamic instability, coupled with adherence to advanced resuscitation protocols, is critical for improving survival in high-risk mediastinal tumor surgeries.

## Introduction

Giant mediastinal tumors(GMTs, diameter ≥ 10 centimeter) (1), which are relatively rare in clinical cases. Due to their large size, it compress the adjacent organs (trachea, esophagus, heart and major blood vessels), significantly impacting the respiratory and circulatory systems, potentially causing respiratory distress, cardiac arrest and other serious complications (2).Surgical removal is the preferred treatment for such tumors. However, Patients with a mediastinal mass are at risk for cardiorespiratory complications in the perioperative period (3).Previous studies have reported that the incidence of postoperative respiratory and circulatory complications in adult patients with mediastinal tumors is 11.4%, which is life-threatening (4). Recent studies on GMTs have made significant progress. A 2022 study analyzing 242 patients with GMTs identified major symptoms such as shortness of breath, cough, and chest pain. The study also highlighted that factors like the malignancy of the tumor, its maximum diameter, and the tumor’s location in the posterior mediastinum were key predictors of postoperative survival (1). Although previous studies have addressed postoperative complications in GMTs, this case uniquely illustrates cardiac arrest triggered by abrupt mediastinal shift and rapid lung re-expansion, compounded by gaps in intraoperative monitoring. This report aims to analyze the multifactorial etiology of cardiac arrest in GMT resections, propose preventive strategies, and contrast this case with existing literature to enhance perioperative guidelines for high-risk mediastinal surgeries.

## Case report

**Figure 1:**
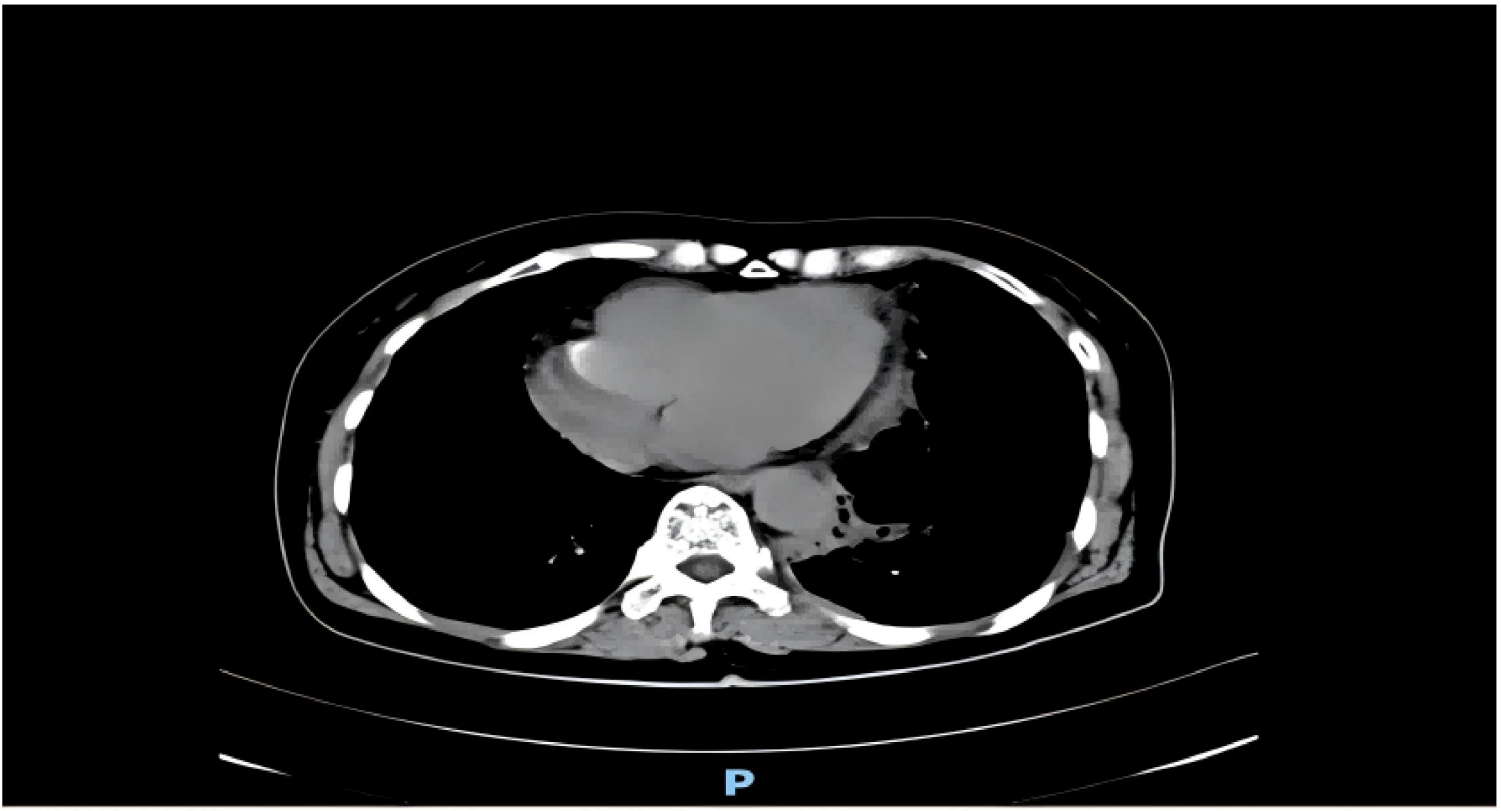
Chest computed tomography of the patient before the operation. There was a massive mass in the anterior mediastinum.(2023 3.24).

**Figure 2:**
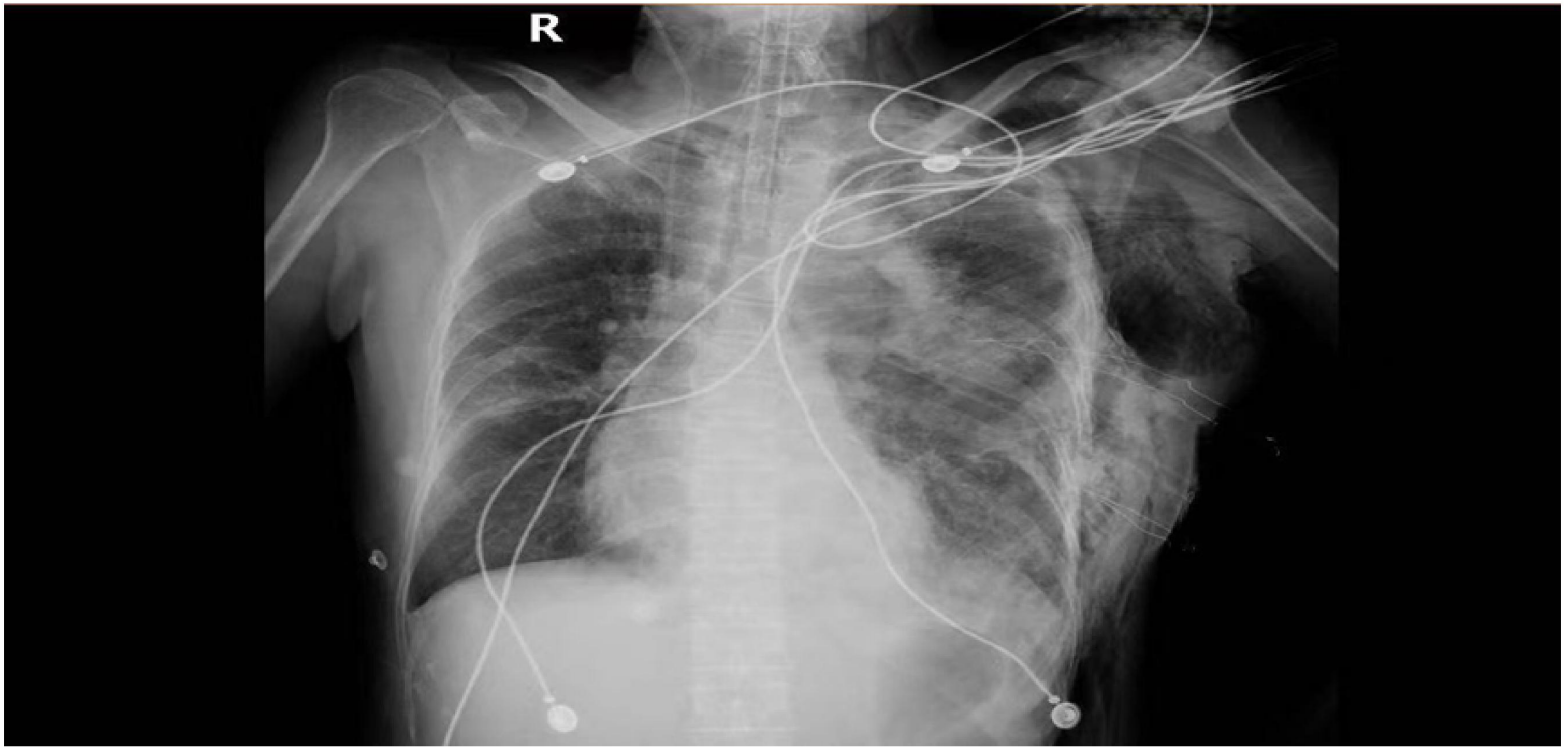
Chest X-ray of the patient 2 hours after the operation. (2023.3.29).

**Figure 3:**
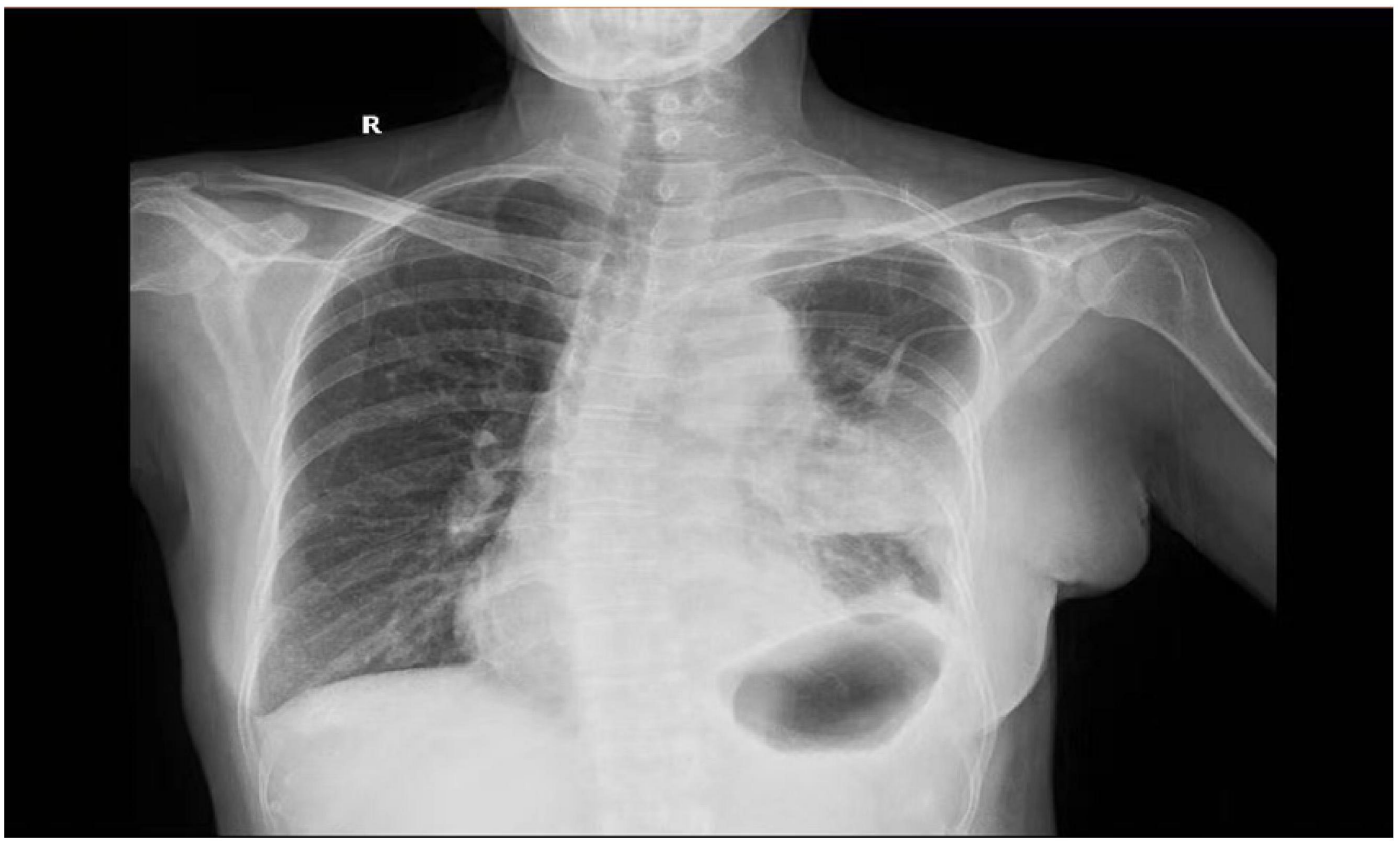
Chest X-ray of the patient half month after the operation. (2023.4.14).

A 54-year-old female with was a history of left anterior mediastinal mass was admitted to Guangxi Medical University Cancer Hospital on March 22, 2024. Lung function showed moderate to severe restrictive pulmonary dysfunction, and cardiac function was normal. On March 29, the patient underwent mediastinal mass resection and wedge resection of the upper lobe of the left lung under general anesthesia. Family history (non-contributory) and social history were abbreviated to focus on relevant medical details. Chest CT showed a massive mass in the anterior mediastinum (**Figure 1**). Differential diagnosis includes: Thymoma, typically located in the anterior mediastinum, though occasionally found in the posterior mediastinum. Patients may present with myasthenia symptoms. CT imaging may show a mediastinal mass, and pathological examination after resection can confirm the diagnosis. Germ cell tumors, often found in the middle mediastinum, usually asymptomatic, though some patients may experience chest pain, tightness, or shortness of breath. CT imaging may reveal a mediastinal mass with uneven density, and elevated serum AFP and βHCG levels may be observed. Pathological examination after surgical resection confirms the diagnosis. Teratoma, generally asymptomatic, with chest CT showing an anterior superior mediastinal mass with uneven density and calcifications. A multidisciplinary team (anesthesiology, critical care) performed risk stratification, identifying susceptibility to mediastinal swing and pulmonary edema. Anesthetic planning prioritized lateral positioning to minimize cardiac compression and invasive hemodynamic monitoring. The operation was removal of about 800ml of cystic fluid and 1500ml of hemorrhage, postoperative hypotension (64/37 mmHg) and slowed heart rate (50 beats/min) were observed. After administration of ephedrine, the blood pressure rose to 115/64 mmHg. 21:00 was transferred to the anesthesia resuscitation room, the blood pressure dropped again (55/36 mmHg), and was urgently transferred to the ICU. no cardiac monitor was carried during the transfer, and the patient’s face was cyanotic, and the carotid artery was not palpated, so he was immediately given cardiopulmonary resuscitation, and was given first-aid measures such as dilatation of fluids and adrenaline. 21:25 cardiac monitor showed Sinus rhythm, heart rate 106 beats/min, oxygen saturation 99%, but not conscious, continue ventilator-assisted ventilation (**Figure 2**). On the 6th postoperative day, he became conscious, and after removing the endotracheal tube, he had difficulty breathing and was reintubated. On postoperative day 12, the tracheal tube was extubated again and high-flow oxygen was used instead. The chest tube was removed on the 14th postoperative day (**Figure 3**), and the patient was transferred to the Department of Thoracic Tumor Surgery, and was discharged from the hospital on the 20th postoperative day. The following is a timeline of disease progression and treatment from the time a patient is transferred to the unit to which they are transferred. The following is a visual timeline of treatment and test results included in the manuscript (**Table 1**).

**Table 1:** Visual timeline of treatment and test results.

Date	Event Description
21:00	Successful resuscitation followed by continued ICU care with mechanical ventilation, fluid resuscitation, and antibiotics for infection(Schuepsin + levofloxacin + vancomycin + fluconazole).
Post-op Days 1–6	Mechanical ventilation and antibiotics for suspected aspiration pneumonia.
Day 6	At 16:05, respiratory distress reoccurred. Changed to Venturi mask for oxygen support. Rechecked blood gas, indicating respiratory failure. Immediate use of non-invasive mask for assisted ventilation.
Day 12	Re-intubated and connected to the ventilator for assisted ventilation.
Day 14	Extubated (removal of endotracheal tube).Transferred back to thoracic surgery for continued treatment.
Day 20	Successfully discharged from hospital.

## Discussion

The patient’s arrest stemmed from abrupt hemodynamic shifts post-tumor removal. Mediastinal swing caused cardiac torsion and reduced venous return, while rapid lung re-expansion precipitated pulmonary edema. These mechanisms align with prior reports of GMT-related complications but are uniquely severe in this case due to delayed recognition of instability during transfer.

### Mediastinal mass syndrome and its implications

Mediastinal Mass Syndrome (MMS) is a rare but serious condition caused by the compression of vital structures in the mediastinum, such as the heart and large blood vessels, by a mediastinal tumor. Symptoms associated with MMS include cough, wheezing, dyspnea (5), syncope, cyanosis, arrhythmias, and in extreme cases, cardiac arrest (CA) (6, 7). The severity of these symptoms can vary depending on the tumor’s size and its impact on surrounding anatomical structures (8). Notably, studies have indicated that postoperative MMS occurs in up to 45.9% of patients undergoing mediastinal tumor resection (9), highlighting the importance of careful preoperative assessment and postoperative monitoring.

In this patient, the preoperative CT scan((**Figure 1**))revealed a massive mediastinal mass, causing chest pain and shortness of breath, which aligned with the diagnosis of MMS. The patient’s hemodynamic stability in the right lateral recumbent position before surgery, coupled with a moderate to severe restrictive ventilation pattern on pulmonary function testing, signaled potential risks associated with surgery (10). Despite these findings, the tumor resection proceeded under general anesthesia due to the decision to prioritize tumor removal given the patient’s preoperative condition and tumor burden.

### Hemodynamic changes and cardiac arrest

During the postoperative transfer, the patient experienced a dramatic hemodynamic collapse, with blood pressure dropping to 64/37 mmHg, which ultimately led to a cardiac arrest. The likely cause of this hemodynamic instability was the release of cardiac compression following the resection of the large mediastinal mass. The abrupt shift in intrathoracic pressure, alongside mediastinal swing, played a significant role. Mediastinal swing refers to the periodic movement of the heart and large vessels due to changes in intrathoracic pressure, usually driven by respiratory movements. These movements can lead to cardiac compression, reducing cardiac output, and may cause compression of major blood vessels such as the vena cava and pulmonary arteries, thereby restricting blood flow. Additionally, the heart’s periodic oscillations during mediastinal swing can induce arrhythmias due to nerve stimulation in the surrounding tissues (2). This pathophysiological mechanism explains why such a significant drop in blood pressure occurred following tumor removal.

Postoperative reexpansion pulmonary edema (RPE) further contributed to the development of cardiac arrest (11). After the tumor was resected, the left lung rapidly reexpanded, causing a restoration of left pulmonary artery pressure and significant hemodynamic shifts. Ultrasound findings revealed left atrial dilatation and reduced left ventricular systolic function, indicating the onset of pulmonary edema (12). RPE typically occurs when the lung reexpands too quickly, leading to fluid leakage from the pulmonary microvasculature. This process can severely impair oxygenation and induce circulatory disturbances. In this patient, prolonged tumor compression likely led to hypoxia in the lung tissue, which, combined with the rapid postoperative reexpansion and increased venous return, triggered the acute pulmonary edema. The interplay of these factors underscores the complexity of managing patients undergoing mediastinal tumor resections and highlights the need for careful postoperative monitoring (13, 14).

### Emergency resuscitation and challenges

The successful resuscitation of this patient underscores the importance of timely intervention in the event of cardiac arrest. The prompt recognition of CA, followed by immediate high-quality chest compressions, was key to saving the patient’s life. This approach is consistent with the 2023 AHA Guidelines (15), which emphasize the importance of early and effective chest compressions in improving survival outcomes. Despite the challenges posed by the patient’s unresponsiveness post-surgery and the absence of monitoring during the transfer, the medical team’s extensive experience ensured a successful emergency response. The patient was stabilized following the administration of antihypertensive drugs and rapid fluid resuscitation.

However, the case also highlighted several critical deficiencies that need to be addressed for future patient safety. The preoperative assessment, though thorough in some respects, failed to fully account for the risks associated with postoperative positional changes and the potential for mediastinal swing during transfer. This oversight led to the sudden drop in blood pressure and contributed to the patient’s cardiac arrest. One potential improvement could be the implementation of a more detailed risk stratification protocol, which includes consideration of the mechanical effects of tumor resection on intrathoracic pressure and subsequent hemodynamic stability.

The lack of continuous monitoring during the transfer from the operating room to the recovery area was another significant gap in patient care. While the patient’s immediate postoperative condition seemed stable, the absence of monitoring during transport hindered the team’s ability to detect early signs of hemodynamic instability. For future surgeries, it is crucial to maintain continuous monitoring during all postoperative transfers, particularly for patients who have undergone major resections like mediastinal tumor removal. A standardized protocol for high-risk transfers (e.g., portable ECG, pulse oximetry) could mitigate this.

### Treatment and postoperative considerations

The choice of maintaining the patient in a right lateral position during surgery was appropriate for minimizing pressure on the heart and large vessels (9). However, the postoperative shift in position was not adequately managed. This change, coupled with the rapid reexpansion of the lung, likely contributed to the cardiovascular instability. Recommendations for future procedures include maintaining lateral positioning throughout the immediate postoperative period to prevent exacerbation of mediastinal swing and associated hemodynamic changes.

Furthermore, the decision to initiate rapid fluid resuscitation after the blood pressure drop was appropriate, but the lack of a timely ultrasound assessment to evaluate circulating volume and the potential for RPE may have delayed appropriate interventions. Preoperative echocardiography and intraoperative transesophageal ultrasound might have better predicted re-expansion risks. Early detection of fluid overload or pulmonary edema through imaging could have helped guide more precise resuscitation efforts and potentially reduced the severity of the patient’s condition.

The patient’s secondary intubation, despite a successful tumor resection, was a direct result of inadequate postoperative ventilation management. The need for prolonged mechanical ventilation (lasting nine days) and subsequent reintubation was indicative of insufficient respiratory assessment and support post-surgery. In retrospect, more comprehensive preoperative and postoperative respiratory assessments, alongside a more detailed respiratory care plan, could have potentially mitigated the need for reintubation.

## Conclusion

This case suggests that perioperative management of patients undergoing surgery for giant mediastinal tumors is challenging, and complications of giant mediastinal tumors are variable and lack specific treatment options. Preoperative imaging and risk stratification must guide anesthetic and surgical planning. Preoperative multidisciplinary consultation is required to adequately assess surgical and postoperative risks and develop contingency plans; Hemodynamic monitoring should persist through all perioperative phases, including transfers. and the patient should be kept in the same lying position as intraoperatively as much as possible after surgery and when transferring the patient, and the patient’s position should be changed gradually after the compressed lungs have reexpanded. This report advocates for protocolized management of GMTs, integrating advanced monitoring and multidisciplinary collaboration to reduce mortality.

## Data Availability

The raw data supporting the conclusions of this article will be made available by the authors, without undue reservation.
